# Smart pooling: AI-powered COVID-19 informative group testing

**DOI:** 10.1038/s41598-022-10128-9

**Published:** 2022-04-20

**Authors:** María Escobar, Guillaume Jeanneret, Laura Bravo-Sánchez, Angela Castillo, Catalina Gómez, Diego Valderrama, Mafe Roa, Julián Martínez, Jorge Madrid-Wolff, Martha Cepeda, Marcela Guevara-Suarez, Olga L. Sarmiento, Andrés L. Medaglia, Manu Forero-Shelton, Mauricio Velasco, Juan M. Pedraza, Rachid Laajaj, Silvia Restrepo, Pablo Arbelaez

**Affiliations:** 1 Center for Research and Formation in Artificial Intelligence, Universidad de los Andes, Bogotá, Colombia; 2 Laboratory of Applied Photonics Devices, École Polytechnique Fédérale de Lausanne, Lausanne, Switzerland; 3 School of Science, Universidad de los Andes, Bogotá, Colombia; 4 Applied Genomics Research Group, Vice Presidency for Research and Creation, Universidad de los Andes, Bogotá, Colombia; 5 School of Medicine, Universidad de los Andes, Bogotá, Colombia; 6 Department of Industrial Engineering, Universidad de los Andes, Bogotá, Colombia; 7 Department of Physics, Universidad de los Andes, Bogotá, Colombia; 8 Department of Mathematics, Universidad de los Andes, Bogotá, Colombia; 9 School of Economics, Universidad de los Andes, Bogotá, Colombia; 10 Department of Biomedical Engineering, Universidad de los Andes, Bogotá, Colombia; 11 Department of Computer Science, Johns Hopkins University, Baltimore, USA

**Keywords:** Computer science, Epidemiology, Machine learning

## Abstract

Massive molecular testing for COVID-19 has been pointed out as fundamental to moderate the spread of the pandemic. Pooling methods can enhance testing efficiency, but they are viable only at low incidences of the disease. We propose Smart Pooling, a machine learning method that uses clinical and sociodemographic data from patients to increase the efficiency of informed Dorfman testing for COVID-19 by arranging samples into all-negative pools. To do this, we ran an automated method to train numerous machine learning models on a retrospective dataset from more than 8000 patients tested for SARS-CoV-2 from April to July 2020 in Bogotá, Colombia. We estimated the efficiency gains of using the predictor to support Dorfman testing by simulating the outcome of tests. We also computed the attainable efficiency gains of non-adaptive pooling schemes mathematically. Moreover, we measured the false-negative error rates in detecting the ORF1ab and N genes of the virus in RT-qPCR dilutions. Finally, we presented the efficiency gains of using our proposed pooling scheme on proof-of-concept pooled tests. We believe Smart Pooling will be efficient for optimizing massive testing of SARS-CoV-2.

## Introduction

COVID-19 is an acute respiratory illness caused by the novel coronavirus SARS-CoV-2^1,2^. It has rapidly spread to most countries, causing 182 million confirmed infections and over 3.9 million deaths as of June 29th 2021^3^. In order to contain the ongoing pandemic, countries have rushed to implement massive testing to control the spread of the disease by identifying and isolating carriers. Massive testing is a fundamental strategy to curb the disease^4^, mainly due to its asymptomatic transmission^5^. Large-scale testing is costly and requires scarce reagents. As there is a global need to make testing more accessible to larger populations, strategies to increase the number of people that can be tested with the same amount of test kits are urgent.

Dorfman proposed pooling methods^6^ to diagnose syphilis among the US military during World War II by combining samples from multiple soldiers in a single test tube. Different pooling strategies, such as Dorfman’s two-stage pooling^6,7^ and matrix pooling^8^, offer higher efficiency gains for different combinations of test sensitivity and disease prevalence.

Existing works have demonstrated that using prior information to exclude samples likely to be positive from pools can increase testing efficiency. In informative group testing, as stated by Bilder et al., the different probabilities of individuals testing positively are exploited by grouping their samples into pools that are more likely to be all negative^9^. McMahan et al. proposed to threshold individuals as “high” or “low risk” and to use risk-specific algorithms while simultaneously identifying pool sizes to minimize the expected number of tests^10^. Taylor et al.^11^ used a complementary microscopic examination of samples in the grouped detection of malaria and Lewis et al.^12^ used clinical information in patients’ forms in the grouped detection of chlamydia and gonorrhea.

Previous studies in group testing aim to reduce the number of tests by finding the optimal design choices such as the pool sizes and the number of stages, and the assignment of specimens to the pools, as explored in^13^ by estimating the probability of disease positivity. Aprahamian et al. proposed to maximize the classification accuracy by reducing the number of false negatives under a budget constraint and evaluated an adaptive risk-based pooling scheme on Chlamydia screening in the US^14^. Additionally, pooling has also been used in genetic sequencing^15,16^ and drug screening^17^, among other applications. In the context of COVID-19, pooling strategies have been explored as potential ways to increase testing capacities. Bloom et al. use simple pooling to detect SARS-CoV-2 through next-generation sequencing instead of the traditional RT-PCR procedure^18^. Libin et al. explore the use of a pooling strategy that groups individuals that belong to the same household^19^. However, the selection of samples to pool for both of these approaches could be improved by using a broader context of the individuals.

**Figure 1:**
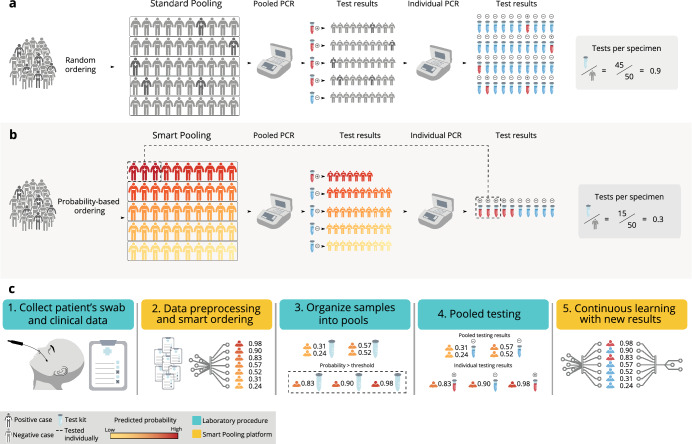
Smart Pooling makes pooling methods efficient even at high disease prevalences. (**a**) In standard Dorfman’s testing methods, samples are pooled randomly. As prevalence increases, the efficiency of pooling without a priori information drops rapidly, making the strategy unviable. (**b**) Smart Pooling tackles this problem by thresholding samples according to the automated predicted risk probability, based on the sociodemographic characteristics. If the risk probability of a sample surpasses the defined threshold, this sample goes directly to individual testing. We arrange samples into pools with similar risk probability. (**c**) The Smart Pooling pipeline directly integrates machine learning with laboratory procedures.

In this paper, we present Smart Pooling, a machine learning (ML) method for optimizing massive testing of SARS-CoV-2. We exploit clinical and sociodemographic characteristics of samples from a retrospective dataset with patient-level information to estimate their posterior probability of testing positive for COVID-19. These characteristics include variables related to their health situation, such as comorbidities, symptoms, date of onset of symptoms, previous contact with confirmed COVID-19 cases, and personal information from individuals such as sex, age, occupation, health system affiliation, and recent travels. As Fig. 1b shows, our method uses the estimated probabilities to arrange samples into pools that maximize the probability of yielding a negative result within the majority of pools. Samples predicted to be positive are tested individually, reducing the number of kits required to diagnose the same amount of individuals. Our method can be used jointly with existing pooling algorithms to enhance their efficiency. Smart Pooling can be thought of as an informative group testing^10^ in which the patients’ probability of positivity is learned from clinical data through state-of-the-art Artificial Intelligence (AI) techniques.

Empirical tests have confirmed the ability of pooled sampling to reliably detect SARS-CoV-2 in a pool comprising one positive and up to 31 negative specimens^20,21^. Moreover, preliminary trials show that pools of 5^22^ and 10^23^ samples can increase efficiency without strongly compromising sensitivity. At the same time, Bayesian non-adaptive pooling schemes demonstrate increased efficiency for low prevalences in experiments in vitro^24^. Pooling strategies could facilitate detection of early community transmission^25^. However, they begin to fail as disease prevalence increases^26^, as shown in Fig. 1a.

In the context of COVID-19, AI-based models have been used to classify the novel virus from its genetic sequencing^27^, to support diagnosis from CT scans^28^, to assist clinical prognosis of patients^29^, and to forecast the evolution of the pandemic^30^. Menni et al.^31^ used regression models trained with patient-reported symptoms and laboratory test results to predict infection. Using these strategies for diagnostics risks compromising sensitivity and confidence. Smart Pooling does not seek to replace current molecular testing but assists it by improving its efficiency.

Smart Pooling is easily adaptable to any pooling procedure. We propose a five-step pipeline between the laboratory procedures and the Smart Pooling analysis. Figure 1c illustrates this process. First, the laboratory acquires samples and complementary data from patients. Secondly, the Smart Pooling platform processes the data and triages the samples. Thirdly, samples are pooled according to this ordering in the laboratory. Then, molecular tests are run on the ordered pools until there is a diagnosis for each sample. Lastly, the laboratory feeds the results of the tested samples into the Smart Pooling platform to improve the model continuously.

**Figure 2:**
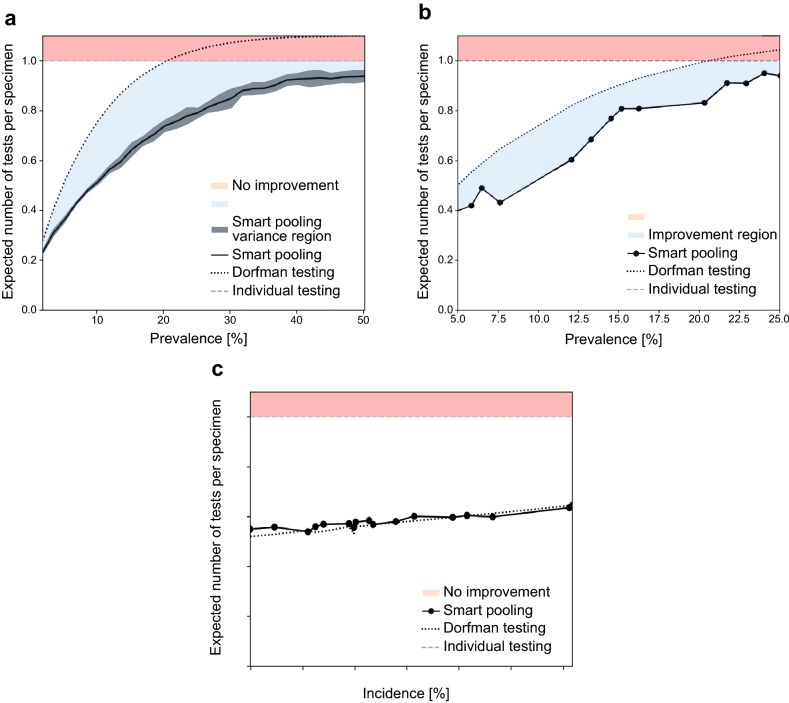
Smart Pooling decreases the expected number of tests per specimen. (**a**) Smart Pooling’s expected number of tests per specimen compared to standard testing methods on Patient Dataset. Efficiency improves by reducing the expected number of tests per specimen. Smart Pooling achieves higher efficiencies than Dorfman’s and individual testing for prevalences of the disease of up to 50%, with a fixed pool size of 10. (**b**) Smart Pooling’s expected number of tests per specimen trained with the coarse metadata from the Test Center Dataset. Efficiency improves by reducing the expected number of tests per specimen. Despite not having detailed patient metadata, Smart Pooling can produce higher efficiencies than Dorfman’s pooling for the simulated prevalence of the disease of up to 25%, with a fixed pool size of 10. (**c**) Expected number of tests per specimen for the proof-of-concept Smart Pooling Implementation. Each point on the graph represents the performance of both methods for a day in the proof-of-concept stage. The x-axis depicts the incidence of the corresponding day. Efficiency improves by reducing the expected number of tests per specimen. Smart Pooling has a similar efficiency compared to Dorfman’s testing since the daily incidence is lower than 10%. However, for every day of the implementation, Smart Pooling has a higher efficiency than individual testing.

Smart Pooling decreases the expected number of tests per specimen to 0.36 and 0.94 at a disease prevalence of 5% and up to 50%, respectively, including a regime in which Dorfman’s testing procedure no longer offers efficiency gains compared to individual testing (see Fig. 2). In this context, we define efficiency as the inverse of the expected number of tests per specimen. Additionally, we calculate the possible efficiency gains of one- and two-dimensional two-stage pooling strategies and present the optimal strategy for disease prevalences up to 25%. We discuss practical limitations to conduct pooling in the laboratory. Pooled testing has been a theoretically alluring option to increase diagnostics coverage since its proposition by Dorfman. Although there are examples of successfully using pooled testing to reduce diagnostics costs, its applicability has remained limited because efficiency drops rapidly as prevalence increases. Not only does our method provide a cost-effective solution to increase the coverage of testing amid the COVID-19 pandemic, but it also demonstrates that artificial intelligence can be complementary to well-established techniques in the medical praxis. To ensure reproducibility and promote further research, we make a portion of our dataset available for public use in the supplementary information.

## Methods

We used an automated Machine Learning (ML) method^32^ to train several models on a retrospective dataset of RT-qPCR tests for SARS-CoV-2, including clinical and sociodemographic information of samples. We used the predictions of the best ML model to simulate the Smart Pooling process. We also found the theoretical optimal pooling protocols for a given prevalence. Then, we evaluated the sensitivity of pooled RT-qPCR SARS-CoV-2 testing for dilutions between 0x and 28.9x. Finally, we executed proof-of-concept experiments of our proposed AI-assisted Dorfman’s pooled testing. This study was approved by the ethics committees at Universidad de los Andes and the Health Authority of Bogotá after the due revision process, and it was performed following the Declaration of Helsinki regulations. As an anonymized retrospective study, the need for informed consent was waived by the ethics committee at Universidad de Los Andes.

### Dataset

The data we used correspond to the molecular tests conducted by Universidad de Los Andes for the Health Authority of Bogotá, Colombia. We tested samples individually following the Berlin Protocol^33^ and the protocol for the U-TOP^TM^ COVID-19 Detection kit from Seasun Biomaterials^34^, before and after April 18th respectively. Our dataset consists of two different data groupings, representing the diversity of available data, the Patient Dataset and the Test Center Dataset. On the one hand, the goal of the Patient Dataset is to capture the epidemiological and social characteristics of individuals that may lead to infection of COVID-19. On the other hand, the goal of the Test Center Dataset is to depict the temporal evolution of the pandemic at a group level. Our work has the ethics committee’s approval at Universidad de Los Andes, and, as an anonymized retrospective study, the need for informed consent was waived.

#### Patient dataset

In this partition of the dataset, we had access to additional information for each sample, such as sex; age; date of onset of symptoms; date of the medical consultation; initial patient classification; information about the patients’ occupation; affiliation to the health system; travels (international or domestic); comorbidities; symptoms; and if they had come into contact with a confirmed or suspected COVID-19 case. We collected 2068 samples with an overall prevalence of 6.91% from April 18th to July 15th 2020. These samples correspond to 1142 male patients (55%) and 926 (45%) female patients. The patients’ ages ranged from 0 to 93 years, with a median age of 36 years. This additional information followed the protocol by the Colombian Public Health Surveillance System (SIVIGILA).

#### Test center dataset

We organized the second group of data according to the test center that collected the sample. We had access to information about the test centers, such as their location, name, the number of positive and total tests per center on a given date (although not daily reports), but no information regarding the individual patients. This dataset included 7162 samples, from 101 test centers, from April 6th to May 25th 2020 with a prevalence of 15.04%.

### Training of the predictors

#### Dataset division

For the Patient Dataset, the distribution of epidemiological and social characteristics of individuals that get tested for COVID-19 can change drastically within a day. We cannot explicitly model the time variations in this dataset because we do not have longitudinal patient data. Thus, the best way to exploit all of the specific information that we have from individuals is to create a two-fold cross-validation scheme, where we use one data fold for training and the held-out fold for evaluating the model. We created the folds using a stratified sampling strategy, where each fold had the same data distribution. However, notice that, even though we trained on a cross-validation scheme, our method can be evaluated in a real-life scenario where we perform daily predictions (see the *Proof-of-concept Smart Pooling Implementation* Section).

For the Test Center Dataset, we modeled the data according to each test center and its progression through time. The prevalence in the test centers evolves with the pandemic’s development. We defined three temporal divisions of the data chronologically for our experiments: the training, validation, and test splits. For validation and testing, we held out five dates to predict in accordance with data availability. To obtain our final results in the test set, we retrained the best model with the training and validation samples, that is, those up to May 7th.

#### Training the models

We used the AutoML library from H2O^35^ to explore multiple ML models and their hyperparameters in the validation sets, namely Random Decision Forests^36^, Generalized Linear Models^37^, Gradient Boosting Machines (GBM)^38^, Fully Connected multi-layer Neural Networks^39^, and Stacked Ensembles. Among the available models, we did not include deep learning algorithms because of the relatively small size of the datasets.

Patient Dataset. We trained our method to predict the label of a sample as positive or negative for COVID-19 by optimizing the Area under the Precision-Recall Curve. For these experiments, we defined a per-sample descriptor in which each feature dimension corresponds to a variable from the patient and location information, previously defined in the *Patient Dataset* subsection. The trained model’s output is a probability for each patient to test positive. We obtained the final prediction via our cross-validation scheme by merging each split’s prediction. For simplicity, in our main results, we used a pool size of 10, and we compare in the Supplementary Fig. 1 this strategy and the optimal strategies per prevalence according to the findings from the *Optimal pooling strategies* Section.

The best model for this task was a GBM^38^ with 30 trees, a mean depth of 9, a minimum of 12 leaves, and a maximum of 22 leaves. We include a comparison of different models in the first section of the supplementary information.

Test Center Dataset We trained our model to predict the fraction of positive tests for a center on the current date. Afterwards, we assigned this value as the sample’s incidence given the test center and date. For these experiments, the descriptors to predict the incidence for a date in the validation and test set included the cumulative tests of each institution up to every date within the time series, i.e., the institution prevalence, and the total number of tests from all the institutions on the corresponding date. In addition, the temporal information was encoded using the current date, relative dates (such as the number of days since the first date on the time series and from the first *n* number of positive tests in each test center), and a variable that captured the distance between two consecutive entries from the same test center. As such, we compute the descriptor’s features by analyzing the relative differences between variables on the last known date and those from the training days in the current difference of days. For a more in-depth description of the *Test Center Dataset*’s training process we refer the reader to the supplementary information.

#### Mathematical formulation

Let $I = \{i_1, i_2, ..., i_n\}$ be the set of *n* samples arriving to a testing facility, and $\mathcal {I}$ be space of all possible samples, $I \subset \mathcal {I}$. To enhance the performance of the Dorfman testing, Smart Pooling follows a similar approach of McMahan *et al.*’s *Optimal Dorfman with Threshold* procedure^10^. Let *f* be an ML model, $f: \mathcal {I} \rightarrow [0,1]$, that computes the individual probability $p = f(i)$ of a sample $i \in \mathcal {I}$ from having the disease. Note that *f* is trained on a set $I_t \subset \mathcal {I}$ that does not intersects *I*, *i.e.* $I \cap I_t = \varnothing$. First, *f* extracts every probability $p_j = f(i_j)$ for all $i_j \in I$ and creates a new ordered set $I' = \{i_1', i_2', ..., i_n'\}$, where $f(i_j') \le f(i_{j+1}')$, $\forall j \in \{1, 2, ..., n-1\}$. Then, given a fixed pool size of *c* and a threshold *T*, we splitted $I'$ into two subsets $I'_{<T} = \{i' | i' \in I' \text { s.t. } f(i') < T\}$ and $I'_{\ge T} = \{i' | i' \in I' \text { s.t. } f(i') \ge T\}$. The samples within $I'_{\ge T}$ are tested individually, and the ones belonging on $I'_{<T}$ are tested on a Dorfman’s pooling fashion. For the latter, we splitted $I'_{<T}$ into $m = \left\lceil \frac{|I'_{<T}|}{c} \right\rceil$ groups, where $\lceil \cdot \rceil$ is the ceil operation. Let $I'_{k} = \{i'_j | i'_j \in I'_{<T} \text { s.t. } (k - 1) c + 1 \le j \le k c\}$, for $k \in \{1, 2, ..., m\}$, be the set of the *k*’th ordered set, with $\bigcup _{k=1}^{m} I'_k = I'_{<T}$. We followed the commonly used Dorfman’s testing on these sets, including the consequent individual testing whether the group test was positive.

The efficiency of the aforementioned process is computed by counting the number of test kits used to generate the results divided by *n*. That is, the efficiency *E* of the set *I*, given a threshold *T*, a pool size *c* and an ML model *f*, is:

1 $$\begin{aligned} E(I, T, c, f) = \frac{1}{|I|} \left[ |I'_{\ge T}| + \sum _{k=1}^{\left\lceil \frac{|I'_{<T}|}{c} \right\rceil } 1 + |I'_k| \mathbb {I}(I'_k) \right] \end{aligned}$$

where $\mathbb {I}(I'_k)$ is equal to one if at least one sample within $I'_k$ is positive, and otherwise is zero. Thus, to determine *T* for the *Patient Dataset* we optimized the efficiency in the training folds. Given the models $f_1$ and $f_2$ trained on the folds $I_{t_1}$ and $I_{t_2}$, respectively, the threshold is:

2 $$\begin{aligned} T_{\text {Patient}} = \underset{\mathcal {T} \in [0,1]}{\mathrm {argmin}} \left[ E(I_{t_1}, \mathcal {T}, c, f_2) + E(I_{t_2}, \mathcal {T}, c, f_1)\right] \end{aligned}$$

For the *Test Center Dataset*, to compute *T* we selected the optimal threshold on the validation set *V*, given the pool size *c* and a model *f*:

3 $$\begin{aligned} T_{\text {Test Center}} = \underset{\mathcal {T} \in [0,1]}{\mathrm {argmin}} \left[ E(V, \mathcal {T}, c, f)\right] \end{aligned}$$

#### Evaluation

We estimated the efficiency gains by calculating the expected number of tests per specimen. When this value is minimized, efficiency is maximized. To estimate the expected number of tests per specimen at different prevalences of the disease, we synthetically produced subsets of the dataset by randomly excluding negative samples. We performed 10 replicates of these experiments and computed the average and the standard deviation. For our experiments, we assumed a perfect sensitivity from the molecular tests. We refer the readers to the work of McMahan et al.^10^ for an analysis of the effects of reduced sensitivity on group testing efficiency.

## Results

### Efficiency gains from smart pooling

We identify that using complementary information to arrange pools can improve the efficiency of testing. Figure 2 show Smart Pooling’s simulated expected number or test per specimen at disease prevalence ranging from 5% up to 50% in the *Patient Dataset* and from 5% up to 25% in the *Test Center Dataset*, with a fixed pool size of 10. We compare our AI-enhanced Dorfman’s testing against its original counterpart and the individual testing procedure. Note that these ranges are higher than those applicable for optimal standard pooling methods. Overall, Smart Pooling’s efficiency outperforms that obtained by simulating Dorfman’s two-stage pooling and individual testing.

**Figure 3:**
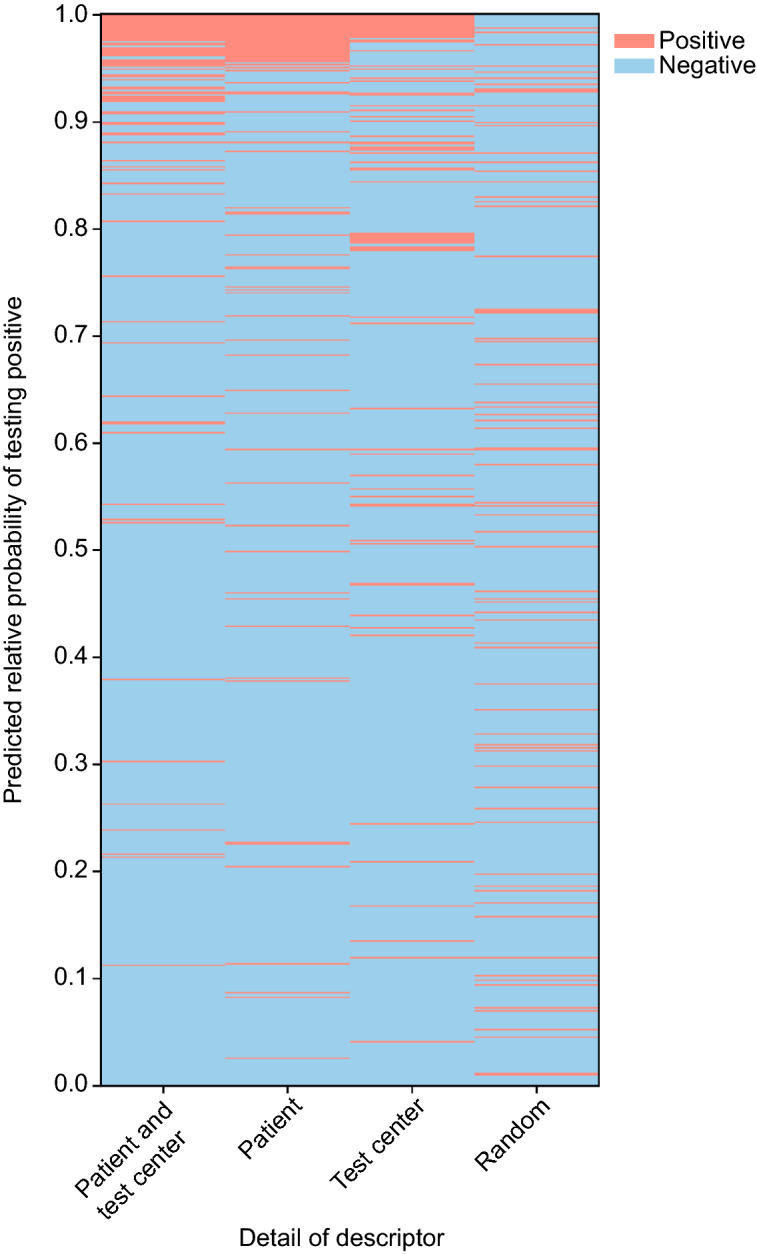
Comparison between Smart Pooling and Random ordering. **Left:** samples arranged by Smart Pooling. **Right:** random ordering of samples. Note that Smart Pooling groups positive samples generating long groups of negative samples, reducing the number of times it is necessary to perform the second stage of Dorfman’s testing, increasing the efficiency.

We rank samples according to the model’s predicted probability of yielding a positive result. Figure 3 shows how the simulated predictions from the AI method have most of the positive samples at the top of the ranking. The key idea of Smart Pooling is that it maintains the prevalence artificially low, even under scenarios with a high overall prevalence of COVID-19, by triaging samples from a priori complementary information before the pooling takes place.

Smart Pooling is not limited to Dorfman’s testing procedure; it can be coupled with multiple pooling strategies and improve efficiency regardless of the strategy. Supplementary Fig. 1 shows Smart Pooling’s simulated expected number of tests per specimen with a fixed pool size of 10, an adaptive pooling strategy based on the optimal strategies explored in the *Optimal pooling strategies* Section, Dorfman’s standard testing, and the individual testing. When coupled with Smart Pooling, these alternatives reduce the expected number of tests per specimen, thus are more efficient than Dorfman’s two-stage pooling and individual testing.

### Optimal pooling strategies

In this section, we find the pooling protocols that maximize efficiency for a given prevalence *p*, assuming a fixed bound *c* on pool size. The efficiency, as mention previously, is defined as the inverse of the expected number of tests per specimen.

We explored the following two kinds of pooling protocols:

1. Dorfman’s pooling protocols^6^: Given *m* samples, we make a single pool with all of them. We denote this protocol by $S_m$.
2. Matrix pooling protocols^8^: Given a collection of $m\times n$ samples, we place them into a rectangular $m\times n$ array. We create pools by combining samples along the rows and columns of this array (for a total of $m+n$ pools). In the second phase, we test each sample at the positive columns and rows intersection individually. We denote this protocol by $S_{m\times n}$.

#### Quantifying pooling efficiency

We define pooling efficiency as our main tool to quantitatively compare different pooling protocols, which depends on the prevalence *p* of the sample. Assuming independence among patients, pooling efficiency can be computed analytically^6,8^:

1. For Dorfman’s pooling $S_m$ the efficiency is given by
  $$\begin{aligned} H(p) = \frac{1}{\frac{1}{m}+1-(1-p)^m} \end{aligned}$$
2. For the matrix pooling protocol $S_{m\times n}$ the efficiency is given by
  $$\begin{aligned} H(p)=\frac{1}{\frac{1}{m} +\frac{1}{n} + p+(1-p)(1-(1-p)^{m-1})(1-(1-p)^{n-1})} \end{aligned}$$

**Figure 4:**
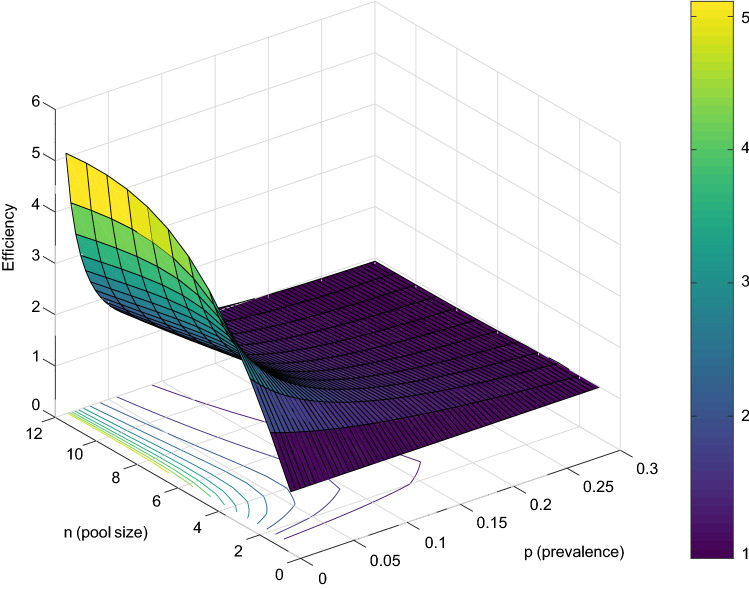
Efficiency of two-stage Dorfman’s pooling as a function of prevalence *p* and pool size *c*.

The efficiency functions above show the key property behind pooling: at low prevalences, efficiency can be considerably greater than one, but as the prevalence increases, *H*(*p*) decreases, with efficiency gains becoming negligible after prevalences around $30\%$. Figure 4 illustrates these efficiency gains (and how they vanish) for two-stage pooling.

#### Optimizing pooling strategies

**Figure 5:**
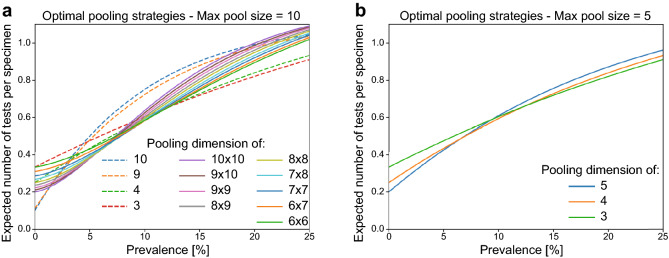
Optimal pooling strategies given a maximum pool size of (**a**) 10 and (**b**) 5.

Practice in the laboratory constrains the maximum pool size. In the context of COVID-19, we will focus on the cases $c=5$ and $c=10$ since these are the most useful in practice (see supplementary information for details). Finding the optimal pooling strategies for a given prevalence equals finding the pooling protocol of maximum efficiency by comparing the values of *H*(*p*) for the different protocols^40^. The maximum efficiency is obtained by minimizing the expected number of tests per specimen. Figure 5 shows the expected number of tests per specimen curves of the best pooling protocols of the form $S_m$ and $S_{m\times n}$ with maximum pool size $c\le 10$ and $c\le 5$. There is an optimal pooling strategy for each prevalence; Table 1 shows the optimal protocols and their respective intervals of optimality when $c = 10$ and $c = 5$ respectively. Even while using optimal protocols, it is clear that efficiency gains quickly disappear as the prevalence increases (effectively vanishing when $p\ge 30.7\%$), which we improve by using Smart Pooling.

**Table 1:** Optimal pooling strategies restricted by a maximum pool size *c*.

Pooling protocol	Prevalence Interval (%)	
	Lower bound	Upper Bound
$$c=10$$		
$S_{10}$	0.00	1.25
$S_9$	1.25	1.375
$S_{10\times 10}$	1.375	4.875
$S_{9\times 10}$	4.875	5.25
$S_{9\times 9}$	5.25	5.875
$S_{8\times 9}$	5.875	6.375
$S_{8\times 8}$	6.375	7.375
$S_{7\times 8}$	7.375	8.125
$S_{7\times 7}$	8.125	9.625
$S_{6\times 7}$	9.625	10.875
$S_{6\times 6}$	10.875	12.125
$S_4$	12.125	12.375
$S_3$	12.375	25.00
$$c=5$$		
$S_5$	0.00	6.625
$S_4$	6.625	12.375
$S_3$	12.375	25.00

Prevalence intervals and their respective optimal pooling strategy. $S_m$ are single pooling protocols and $S_{m\times n}$ are matrix pooling protocols.

### Sensitivity experiments in diluted samples

To test for possible sensitivity reduction from sample dilution in a realistic setting, we performed the following experiment: We diluted a positive sample successively $1.4\times$ ten times in negative samples, from an expected $C_t$ of  38 to  43, in 8 replicates. The diluted samples were processed using the U-TOP COVID-19 Detection Kit (SeaSun Biomaterials)^34^ with the recommended parameters, except that we increased the number of cycles to 43. The average $C_t$ (for the wells that give a reading) increases with dilution but, deviates from the expected value (the observed one is lower), and the number of wells with no reading increases from none, in the initial dilutions, to most, in the higher dilutions. We show the expected average $C_t$ and fraction of wells with no reading for markers N and ORF1ab in Table 2.

**Table 2:** Expected Ct, average $C_t$, and the fraction of wells with no reading for markers N and ORF1ab for successive dilutions of the same sample.

Dilution	ORF1ab			N		
	Expected $C_t$	Average $C_t$	Fail fraction	Expected $C_t$	Average $C_t$	Fail fraction
0X	38.4	39.0	0.00	38.0	37.5	0.00
1.4X	38.9	39.0	0.13	38.5	38.0	0.00
2X	39.4	40.1	0.00	39.0	38.5	0.00
2.7X	39.9	40.5	0.00	39.5	38.9	0.00
3.8X	40.3	40.9	0.63	39.9	39.3	0.13
5.4X	40.8	42.1	0.67	40.4	39.9	0.33
7.5X	41.3	41.6	0.50	40.9	399.0	0.50
10.5X	41.8	–	1.00	41.4	40.2	0.57
14.8X	42.3	42.0	0.88	41.9	40.6	0.75
20.7X	42.8	41.3	0.88	42.4	39.4	0.75
28.9X	43.3	41.7	0.88	42.9	40.3	0.88

We combined the data for both genes and linearly interpolated it to convert this value into an estimate of the number of false negatives. We tabulated the measured $C_t$s of the positive samples from 20,000 tests (2678 samples) and the expected $C_t$s for different dilutions. Assuming the threshold $C_t$ for testing individual samples in a pool is adjusted according to the dilution, the number of samples that would give no reading if in a pool with only negatives for a given dilution can be estimated by convolving the distribution of $Ct_s$ with the failure rate for the resulting $C_t$s after dilution.

The estimate is an upper bound since the calculations are performed for each marker gene separately. The criterion used in practice is to consider a sample positive if $C_t$ is below 38. The latter does not consider the probability that the sample could be in a pool with other positives. For our experiment, we limited the number of cycles to 43. However, a higher number of cycles could give a better performance for more diluted samples. Furthermore, pooling schemes that include the same sample in multiple pools, such as the 8x2 scheme, are less susceptible to this type of error. Table 3 shows the false-negative rate, obtained as explained for single pools of up to 16 samples for markers N and ORF1ab.

**Table 3:** Estimated upper bound for the error (false negative) rate for single pools of up to 16 samples for markers N and ORF1ab.

Dilution	Error rate ORF1ab (%)	Error rate N (%)
$2\times$	0.2	0.2
$3\times$	0.2	0.2
$4\times$	0.6	0.5
$5\times$	2.6	1.5
$6\times$	2.7	2.6
$7\times$	3.5	3.2
$8\times$	4.3	3.9
$9\times$	5.0	4.4
$10\times$	5.4	4.8
$11\times$	6.4	5.8
$12\times$	7.0	6.4
$13\times$	7.6	7.0
$14\times$	8.3	7.6
$15\times$	9.0	8.2
$16\times$	9.7	8.7

### Proof-of-concept smart pooling implementation

This section describes the deployment of Smart Pooling in the GenCore laboratory of the Universidad de Los Andes in Bogotá, Colombia. The project COVIDA, an active surveillance program, provides samples that we use to implement Smart Pooling. COVIDA’s two main objectives are: first, to trace new cases of infection with an active surveillance strategy. Second, the project supports decision-makers by providing relevant information for developing public health policies in Bogotá. Our proof-of-concept experiment in the GenCore laboratory lasted seven days. On average, GenCore tests 300 samples from COVIDA every day. More than 4700 tests were processed during our experiment using the Smart Pooling methodology, and 2012 kits were saved using Smart Pooling instead of individual testing.

For this proof-of-concept stage, the GenCore laboratory implemented Smart Pooling with a pool size of 2. Taking into account that in this stage, we use scarce laboratory resources (equipment and personnel), it was not feasible to evaluate the performance of Dorfman’s testing in the exact same samples for a real-life comparison with Smart Pooling. However, we simulated the theoretical performance of Dorfman’s testing for each day’s samples in the proof-of-concept.

Figure 2 shows the comparison between the performance of Smart Pooling’s results after implementation and Dorfman’s testing simulation. Each point on the graph represents the expected number of tests per specimen for a day in the proof-of-concept stage. The x-axis depicts the incidence of the corresponding day. We show that the performance of Dorfman’s testing is comparable with Smart Pooling; these results are expected due to the low prevalence of the samples in the COVIDA project. The prevalence in the samples is lower than 10% as a result of the project’s active surveillance nature. Nevertheless, implementing Smart Pooling provides further benefits such as an epidemiological overview of the daily state of the pandemic and clear identification of high-risk patients to prioritize the processing of their samples. We also demonstrate that the performance of Smart Pooling follows our initial observation, which pointed out that, regardless of the prevalence, Smart Pooling has higher efficiency than individual testing. Even though the proof-of-concept was done under a limited pool size of 2, our results prove that Smart Pooling can be successfully implemented in laboratories, and it can be easily scaled up for other testing facilities with capacities of performing pooling with larger pool sizes.

To increase the usefulness of Smart Pooling in a lab context, we have to consider some practical implications. First, the samples might arrive at different times in the lab, and it would not be helpful to wait until all the samples from a day are ready to use Smart Pooling. With this problem in mind, we designed our methodology to be adaptable to any amount of samples and to take less than a minute to perform inference. Thus, the lab would perform tests using the Smart Pooling procedure multiple times in a day, depending on their samples’ arrival time. Second, the samples might belong to different collection containers. Since pooling is performed after RNA extraction, the samples will already be organized in a PCR plate. Choosing a specific location for each pool combination might be tedious and time-consuming for the lab technicians. To solve this problem, we devised a strategy that automates choosing the best sample combination for a pool, taking into account the constraint that they should be either in the same row for different columns of the plate or in the exact same location for different plates. This automated solution allows the lab to perform pooling in a more practical way with an eight-channel pipette.

## Discussion

Our computational experiments show that, regardless of the level of granularity of the data available (Fig. 2), Smart Pooling obtains efficiency gains for all simulated prevalences up to 25% for the *Test Center Dataset* and for the *Patient Dataset* up to 50% when comparing against the standard Dorfman’s testing. For instance, with Smart Pooling at a prevalence of 10% and an expected number of tests per specimen around 0.5, even with coarse data in the test center approach, the estimated number of patients that could be tested with the same number of tests is doubled compared to individual testing. These results show that Smart Pooling could be viable in various settings and does not depend on the availability of rich complementary data.

Figure 3 illustrates the working principle of Smart Pooling: it enhances efficiency by artificially reducing the incidence in the samples arranged in pools by testing individually the samples most likely to yield a positive result.

The machine learning model takes advantage of complementary data to compute the probability that the sample results positively. For the *Patient Dataset*, this could come from reported symptoms or the knowledge that the patient belongs to a particular group (for instance, being a health worker). For the *Test Center Dataset*, the machine learning model could be exploiting underlying correlations in the samples^41^ that could be related to the location the samples were taken. The center where the samples are taken could act as a proxy for sociodemographic characteristics of the patients since it could be a location close to their workplace or home. Additionally, COVID-19 outbreaks are usually related to specific geographical regions due to the transmission nature of the virus. Therefore, the model could learn and exploit the underlying correlations present in the test center location to output better predictions. In other words, Smart Pooling could be seen as the assembly of pools with correlated samples where the Dorfman’s protocol’s independence assumption in the underlying binomial distribution no longer holds (within the pool of *correlated* samples)^41^. Samples in this dataset were acquired during strict measures limiting mobility in the city of Bogotá. People tested at the same center likely shared epidemiological factors. The model could also be learning the different probability distributions of samples being positive in various city locations.

### Smart Pooling enhances but does not replace molecular testing

Smart Pooling uses AI to enhance the performance of well-established diagnostics. It demonstrates that data-driven models can complement high-confidence molecular methods. Its robustness to the variability of the available data, prevalence and model performance, and its independence of pooling strategy, make our work compelling to apply at large scales. Additionally, Smart Pooling’s continuous learning should make it robust to our understanding of the pandemic and its evolution.

### Smart Pooling could ease access to massive testing

This pandemic has presented challenges to all nations. As the number of infected people and contagion risk increases, more testing is required. However, the supply of test kits and reagents cannot cope with the demand, with most countries not able to perform 0.3 new tests per thousand people^3^. Adopting Smart Pooling could translate into more accessible massive testing. In the case of Colombia, this could mean testing 50,000 samples daily, instead of 25,000, in mid-July 2020^42^. If deployed globally, Smart Pooling truly has the potential of empowering humanity to respond to the COVID-19 pandemic and save thousands of lives. It is an example of how AI can be employed to bring social good.

## Supplementary Information

Supplementary Information.

## Data Availability

The Test Center Dataset used during the current study is available at the Smart Pooling repository. Patient Dataset belongs to a Colombian government entity and given the confidentiality agreements it is not publicly available.

## Code availability

The main code to reproduce our results is available at the Smart Pooling repository. More information can be found at the Smart Pooling website.
