# Supplementary material for: Smart pooling: AI-powered COVID-19 informative group testing
Source: Sci Rep. 2022 Apr 20;12:6519. doi: 10.1038/s41598-022-10128-9 (PMC9020431; doi:10.1038/s41598-022-10128-9)
Supplement: Supplementary file 1 — Supplementary Information. [file 41598_2022_10128_MOESM1_ESM.pdf]

Smart Pooling: AI-powered COVID-19 Informative Group Testing - Supplementary Material

María Escobar[1], Guillaume Jeanneret[1], Laura Bravo-Sánchez[1], Angela Castillo[1], Catalina Gómez[11,1], Diego Valderrama[1], Mafe Roa[1], Julián Martínez[1], Jorge Madrid-Wolff[2], Martha Cepeda[3], Marcela Guevara-Suarez[4], Olga L. Sarmiento[5], Andrés L. Medaglia[1,6], Manu Forero-Shelton[7], Mauricio Velasco[8], Juan Manuel Pedraza-Leal[7], Rachid Laajaj[9], Silvia Restrepo[4], and Pablo Arbelaez[1,10,*]

[1]Center for Research and Formation in Artificial Intelligence, Universidad de los Andes, Bogotá, Colombia
[2]Laboratory of Applied Photonics Devices, École Polytechnique Fédérale de Lausanne, Lausanne, Switzerland
[3]School of Science, Universidad de los Andes, Bogotá, Colombia
[4]Applied genomics research group, Vicepresidency for research and Creation, Universidad de los Andes, Bogotá, Colombia
[5]School of Medicine, Universidad de los Andes, Bogotá, Colombia
[6]Department of Industrial Engineering, Universidad de los Andes, Bogotá, Colombia
[7]Department of Physics, Universidad de los Andes, Bogotá, Colombia
[8]Department of Mathematics, Universidad de los Andes, Bogotá, Colombia
[9]School of Economics, Universidad de los Andes, Bogotá, Colombia
[10]Department of Biomedical Engineering, Universidad de los Andes, Bogotá, Colombia
[11]Department of Computer Science, Johns Hopkins University, Baltimore, United States
[*]pa.arbelaez@uniandes.edu.co

ABSTRACT

Comparison of prediction models

Table S1. **Prediction models for the Patient Dataset**

Method	Area under PR curve	$F1_{max}$
Gradient Boosting Machine (GBM)	0.45	0.51
Distributed Random Forest (DRF)	0.42	0.49
Naïve Bayes Classifier (NBC)	0.07	0.13
Support Vector Machine (SVM)	0.08	0.13

We compare the performance of four different types of prediction models on the training folds of the Patient Dataset. First, GBM is the best performing model according to the AutoML model and hyperparameter search. This result can be confirmed in supplementary Table S1 with an AUCPR of 0.45 and a maximum F1 measure of 0.51. GBM works by combining multiple decision trees into ensembles. Second, Distributed Random Forest (DRF) also combines decision trees but this combination is done more simply by averaging or choosing the majority at the end of the training process. Thus, the performance of DRF is lower but in the same order of magnitude as GBM, meaning that using either model for Smart Pooling would give satisfactory results. On the other hand, models like Naïve Bayes Classifiers or Support Vector Machines fail to grasp the underlying relationships in the data and give low AUCPR and maximum F1 results.

Model Interpretability

We perform an interpretability analysis to understand further the predictions of the best model for the patient dataset. Since our primary model is a Gradient Boosting Machine, we can calculate each variable's relative influence on splitting decision trees. Thus, we can determine which variables are more relevant to the model during the prediction process. We grouped the variables into seven categories representing the patient's context. First, the COVID-19 related category describes variables that directly respond to someone having COVID-19. The occupation category encompasses questions about the patient's

Table S2. Importance ranking for the top ten variables of the Patient Dataset

Importance ranking	Variable	Category
1	is in tracking	COVID-19 related
2	traffic origin	Occupation
3	alterations in the senses of smell/taste	Symptoms
4	occupation	Occupation
5	goes into tracking	COVID-19 related
6	place of residence	Geographical
7	number of people with whom you live	Familiar
8	neighborhood of residence	Geographical
9	stratum	Social
10	population group	Social

workplace. The geographical category includes information of a patient's location, such as their place of residence. The familiar category constitutes variables regarding the patient's family and living conditions. The social category includes personal and socioeconomic information. The healthcare category includes variables regarding the patient's interaction with their healthcare provider. Finally, there is a specific category to report the patient's symptoms.

Through the interpretability analysis, we were able to identify the ten most important variables for the model. Table S2 shows the importance ranking for each variable and their corresponding category. Although most of the patient's questionnaire is based on identifying multiple symptoms, the model does not consider this information as the most important, except for alterations in the sense of smell or taste. In contrast, the most important variable is if the patient is already being tracked by the health entity. The importance of this variable is consistent with real-life occurrences since only patients that are currently positive for COVID-19 are being tracked. Thus, the model learns that if a patient is in tracking, they probably have had COVID-19 and are still in recovery. The same analogy is accurate for patients that go into tracking after taking the questionnaire. Another important factor that the model considers is the patient's occupation since some occupations might be exposed to the virus because they require more interactions with others. Other relevant variables that the model takes most into account are related to the sociodemographic context of the patient. Figure S1 shows the relative importance that each category has for the model. Although the symptoms category includes the most variables, the model considers it 5% less important than COVID-19 related information. This analysis allows us to increase the explainability of our model and the confidence of using it in a real-life scenario.

Model training of the Test Center Dataset

We had information on the number of positive and total tests on a given date for each test center, but we did not have daily reports. Thus, we explicitly modeled the data as a sparse time series. We trained machine learning models to predict the fraction of positive tests for a center on the current date. Afterward, we assigned this value as the incidence of each sample from a test center on a given date. We sorted the tests by decreasing incidence, simulated a Dorfman testing protocol, computed the number of tests used, and calculated the efficiency of Smart Pooling. The best model for this task was a Gradient Boosting Machine (GBM)[1] with 50 trees, a constant depth of 5, a minimum of 12 leaves, and a maximum of 29 leaves.

To predict the incidence for a date in the validation or test set, we defined a descriptor calculated from the available training data for each testing center. We included as features the cumulative tests of each institution up to every date within the time series (*i.e.* the institution prevalence), and the total number of tests from all the institutions at the corresponding date. To include the temporal information, we defined as features the date in YY-MM-DD format and the relative date in the number of days since the first date on the time series. Additionally, we created a variable that we named *gap*, which encodes the distance between two consecutive entries from the same test center. In particular, the gap encodes the distance between the last training date and the date to predict during the testing stage. The gap provided the model with an estimate of the analyzed time window.

As such, we computed the features of the descriptor by analyzing the relative differences between variables on the last known date and those from the training days in the current gap. These gap features comprise the cumulative number of tests, total tests, number of positive samples for each institution at the last available date, and the corresponding incidences. We complemented the descriptor with features that indicate the number of days, relative to the current prediction date,

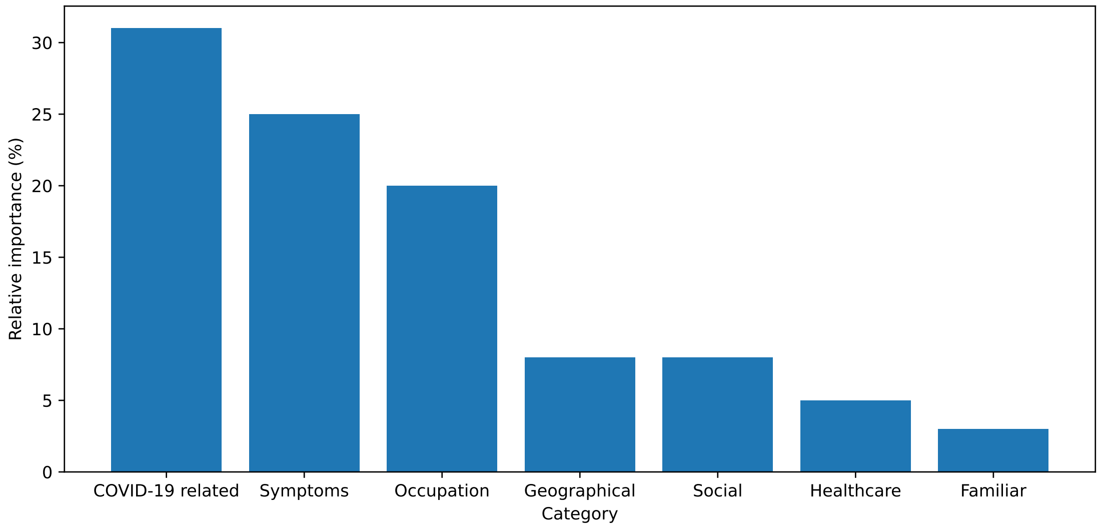

Figure S1. Relative importance of each category.
The model assigns a relative importance to every variable and we aggregate the value according to the category that it belongs to. Note that the model assigns a higher relative importance to variables that are directly related with COVID-19 outcomes.

from the first n number of positive tests in each test center. For our experiments we defined $n = 1, 5, 10, 100, 500, 1000$. The rationale behind this feature was to provide the model with a temporal encoding of the incidence's evolution. We adapted these features from a public kernel from Kaggle's COVID-19 Global Forecasting Challenge, available at https://www.kaggle.com/rohanrao/covid-19-w3-lgb-mad/notebook.

We performed the training of our models in two stages. In the first training phase, we used the validation subset's performance as the criterion to select the best model. We removed the test centers that did not have sufficient dates for the validation split but included them back for the second phase. Lastly, we obtained our final model by retraining the best model using all available training data and evaluated it on the test split.

Practical considerations for implementation

In the specific context of SARS-CoV-2, there are several incentives to implement pooling. The current shortage of reagents is most significant, especially in developing countries that do not produce these and have limited stocks. Additionally, these methods can increase throughput if implemented correctly, another motivation for developing countries and locales with a limited number of certifiable qPCR machines. Finally, through the reduced use of reagents and increased throughput, these methods can reduce costs and motivate healthcare providers.

Here we considered Dorfman and array testing algorithms because they are easily compatible with a manual pooling implementation. Although it is possible to find the optimal algorithm for pooling at a particular prevalence, it may be cumbersome to implement all the protocols. Fortunately, most of the pooling algorithms are relatively robust over a broader range than that in which they are optimal, so implementing some should increase efficiency without added complexity. Different algorithms will also affect tip used as well as plating time. Filter tips have been in short supply since the beginning of the pandemic[2], and some of the pooling protocols increase efficiency at the expense of increased tip use. Plating time will depend on both the pooling algorithm and the experience of the personnel doing the pooling. Each lab has to adapt the specifics of the protocol for their needs.

Successful implementation of PCR-based pooling requires understanding the limitations of the method and performing viability tests. Although it is possible to pool reactions that include primers that detect the virus only when it is present, it is not possible, to our knowledge, to pool the RNAse P positive control. This phenomenon occurs because the samples are positive unless a problem has occurred with the sample or the RNA extraction; it involves finding a negative among positives where samples have variable RNA content and an exponential amplification step. In our implementation, the two reactions are separate. The RNAse P control is performed on a different plate, using a faster PCR protocol (1.5 hours instead of 2.5 hours). In kits for which the specific primers and the control are multiplexed, for example, it is not possible to ensure the presence of RNA when pooling.

Sensitivity must also be taken into account when implementing pooling. Each two-fold dilution increases the Ct value by 1 unit ($1\Delta Ct$), on average. Some have detected dilutions up to 32-fold[3], but only for samples with average Ct, not for samples close to the detection limit. It is necessary to calibrate the dilution process in each lab since it depends on the kit, machine, and operators. Another limit for the size of the pools is the total reagent volume in each pool. Our plates can handle $10\mu l$ of a sample, while other plate-kit combinations may handle just $5\mu l$. Depending on the operators, pipetting volumes under $1\mu l$ may cause quality control problems. Since we rely on volunteers for our testing, we only considered pools of up to 10, which also keeps the maximum Ct of the first round below 42, the limit of cycles in our machine. Additionally, we increased the cutoff in Ct for the first round from 38 to 38+ ΔCt at this dilution, from the calibrations. This modification may increase the number of false positives in the first round, but since the second round samples are sampled individually, the standard cutoff can be used to eliminate potential false positives from the first round.

According to the FDA, there are currently two approaches to perform patient specimen pooling. One of them is the sample/media pooling, which is performed from the aliquots of transporting media, each containing one patient sample. The other is when each transportation media contains multiple swabs from various patients[4]. In that manner, sample/media allow pooling after RNA extraction while swab pooling implies an RNA extraction and RT-PCR in conjunction pooling strategy. Both strategies have different impacts on the tests' sensibility and maximum pooling size. However, this does not imply that the technical viability of the diagnostic test is compromised[5,6]. Pooling before RNA extraction is an attractive option since it reduces reagent use, but we did not implement it for several reasons. The first is that the expected increase in Ct of 5[7] was above the practical limits for our machine and kit combination. Secondly, it is not possible to flag poorly taken samples with no RNA; for the same reasons, it is not possible to pool the RNAse P control. This phenomenon is due to a lower concentration of RNA material in the total aliquot volume of the sample to be analyzed. In contrast, on a pooling after RNA extraction strategy due to a higher initial concentration of RNA material, the sensibility of a test is higher, allowing a maximum pool size of up to 16-32 samples[3]. Due to this phenomenon, several laboratories and pooling strategies for screening and controlling other viral infections[8] use a pooling after RNA extraction strategy. Finally, we received heterogeneous samples of the upper and lower respiratory tracts, resulting in false negatives when pooled together. It is likely that implementing pooling before RNA extraction is possible with other kits or more homogeneous samples.

Test Center Data

We provide the training data for the Test Center experiments on the file named `Test_Center_Dataset.xlsx`. This dataset contains the information that we received from each test center and the PCR results. Column "Sample ID" refers to the unique code to identify each sample. Column "Received Date" has the date on which the test center received the sample. Column "Institution" has the address of the test center. Finally, column "Result" contains the final result for each sample. The rows represent new entries, and they are independent of one another.

Effect of pooling strategy on efficiency

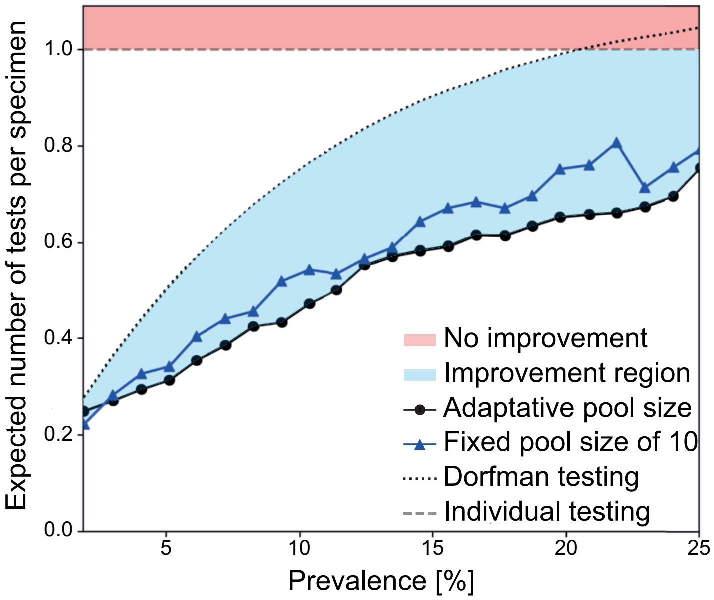

Figure S2. Smart Pooling expected number of tests per specimen of different pooling strategies at individual patient level.
Regardless of the pooling strategy, Smart Pooling obtains superior efficiencies compared to Dorfman testing for prevalences up to 25% by reducing the expected number of tests per specimen. The efficiency is further improved when combining Smart Pooling with the optimal protocols of Adaptive pooling.

References

[1.] Natekin, A. & Knoll, A. Gradient boosting machines, a tutorial. *Front. Neurorobotics* **7**, 21 (2013).

[2.] CNN, Devine, C., Griffin, D. & Kuznia, R. Shortage of standard health supplies is 'a huge problem'. Library Catalog: edition.cnn.com.

[3.] Yelin, I. *et al.* Evaluation of COVID-19 RT-qPCR Test in Multi sample Pools. *Clin. Infect. Dis.* DOI: 10.1093/cid/ciaa531 (2020). Ciaa531, https://academic.oup.com/cid/article-pdf/doi/10.1093/cid/ciaa531/33524991/ciaa531.pdf.

[4.] Food, U., Administration, D. *et al.* Pooled sample testing and screening testing for covid-19 (2020).

[5.] Ben-Ami, R. *et al.* Large-scale implementation of pooled rna extraction and rt-pcr for sars-cov-2 detection. *Clin. Microbiol. Infect.* **26**, 1248–1253 (2020).

[6.] Lohse, S. *et al.* Pooling of samples for testing for sars-cov-2 in asymptomatic people. *The Lancet Infect. Dis.* **20**, 1231–1232 (2020).

[7.] Torres, I., Albert, E. & Navarro, D. Pooling of nasopharyngeal swab specimens for sars-cov-2 detection by rt-pcr. *J. Med. Virol.* **92**, 2306–2307 (2020).

[8.] Pilcher, C. D. *et al.* Real-time, universal screening for acute hiv infection in a routine hiv counseling and testing population. *Jama* **288**, 216–221 (2002).
